# Assessment of delirium in intensive care using the CAM-ICU

**DOI:** 10.1186/cc9757

**Published:** 2011-03-11

**Authors:** R Shetty, K Reid

**Affiliations:** 1 BHR Hospitals, London, UK

## Introduction

Delirium remains a common but poorly diagnosed condition in the ICU [1]. Delirium is an independent predictor of cognitive decline and mortality [2]. The aims of this audit were: to measure the incidence of delirium in our unit; to consider the practicalities of using the CAM-ICU; whether a positive CAM-ICU test would change management; and the attitude of senior intensive care staff regarding the usefulness of CAM-ICU.

## Methods

The CAM-ICU was used for 5 weeks in a mixed general ICU (14 beds) at Queen's Hospital, Romford. Patients were included into the study after 24 hours of admission; they were tested once daily. If the test was positive, a senior physician responsible for the patient's care was asked whether they would change the management of the patient. A survey was conducted to understand the attitude of intensive care consultants regarding the usefulness of the CAM-ICU test.

## Results

**Figure 1:**
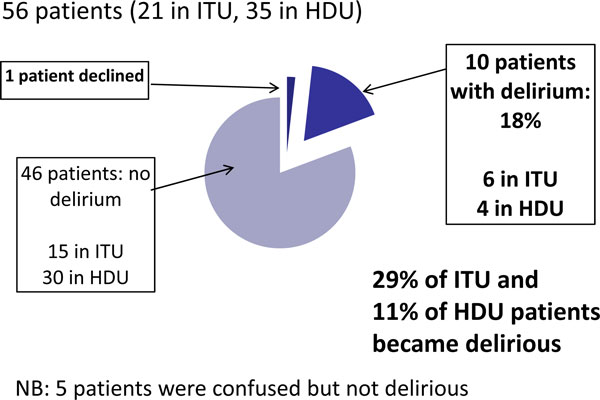
**What is the incidence of delirium?**.

**Figure 2:**
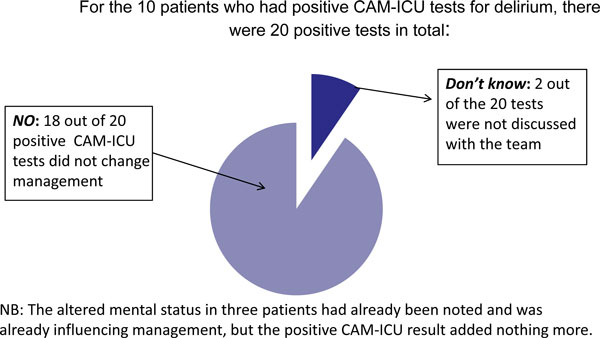
**Do positive CAM-ICU tests change management?**.

Fifty-six patients were included, 10 of which tested positive for delirium (17.9%). Seven were found to be delirious within the first 48 hours of admission. Eight patients had just one episode of delirium. Average length of delirium was 1.75 days. On no occasion did a positive CAM-ICU test result in a change of management. We were unable to assess 22% of patients because they were too sedated (8), not cooperative (7) or for other reasons (8). Surprisingly the survey revealed that more than 75% of the consultants believed a positive CAM-ICU test would result in change in the management of the patient. See Figure 1 and 2.

## Conclusions

The incidence in our unit was lower than in other studies. Daily assessment with the CAM-ICU had no effect on management. It is possible to implement use of the CAM-ICU daily after a short period of training. There is a difference in attitude and practice in senior staff with regard to use of the CAM-ICU. As most cases are short lived and occurred in the first 48 hours, prevention should be emphasized before admission to critical care.
